# Adaption of FMDV Asia-1 to Suspension Culture: Cell Resistance Is Overcome by Virus Capsid Alterations

**DOI:** 10.3390/v9080231

**Published:** 2017-08-18

**Authors:** Veronika Dill, Bernd Hoffmann, Aline Zimmer, Martin Beer, Michael Eschbaumer

**Affiliations:** 1 Institute of Diagnostic Virology, Friedrich-Loeffler-Institut, Südufer 10, 17493 Greifswald-Insel Riems, Germany; veronika.dill@gmx.de (V.D.); bernd.hoffmann@fli.de (B.H.); martin.beer@fli.de (M.B.); 2 Merck KGaA, Merck Life Sciences, Upstream R&D, Frankfurter Straße 250, 64293 Darmstadt, Germany; aline.zimmer@merckgroup.com

**Keywords:** foot-and-mouth disease virus, BHK21, susceptibility, resistance, serotype Asia-1, adaption, suspension cells, adhesion, receptor, antigen production

## Abstract

Foot-and-mouth disease virus (FMDV) causes a highly contagious disease with catastrophic economic impact for affected countries. BHK21 suspension cells are preferred for the industrial production of FMDV vaccine antigen, but not all virus strains can be successfully propagated in these cells. Serotype Asia-1 is often affected by this phenomenon. In this study, the Asia-1 strain Shamir was used to examine viral, cellular and environmental factors that contribute to resistance to cell culture infection. Cell media composition, pH and ammonium chloride concentration did not affect Asia-1 differently than other serotypes. Virus replication after transfection of viral genome was not impaired, but the adhesion to the cells was markedly reduced for Asia-1 in comparison to serotype A. The Asia-1 Shamir virus was successfully adapted to grow in the resistant cells by using a closely related but susceptible cell line. Sequence analysis of the adapted virus revealed two distinct mutations in the capsid protein VP1 that might mediate cell attachment and entry.

## 1. Introduction

In spite of international control efforts, foot-and-mouth disease virus (FMDV) is still widespread in the Middle East, Asia and Africa, where it causes severe disruptions of livestock production and trade [1]. FMDV comprises seven distinct serotypes, namely serotype A, O, C, Southern African Territories (SAT) 1–3 and Asia-1, and a previous infection with one serotype does not protect against an infection with any of the other six [2]. To fight this highly contagious disease, inactivated vaccines are produced at a large scale. The dominant cell line for industrial production of FMDV vaccine antigen is baby hamster kidney-21, clone 13 (BHK21C13) by MacPherson and Stoker [3], adapted to grow in suspension by Capstick et al. [4], and used in large fermenters as first described by Telling and Elsworth [5]. Unfortunately, BHK21 cells have some phenotypic features that are detrimental for production of FMDV antigen. Along with alterations in cell ploidy and a down-regulated surface expression of particular integrins correlated with the loss of actin stress fibers (especially in suspension) [6,7,8], BHK cells vary in their susceptibility for different FMDV strains [9,10]. Furthermore, the ability for FMDV infection can get lost on repeated subculturing [9,11].

To test the susceptibility for FMDV of a cell line, serotype Asia-1 viruses may serve as an indicator because this serotype has the most difficulties to grow in different BHK cell lines. A cell line that supports growth of Asia-1 usually is susceptible to all other serotypes of FMDV [9,10].

While some studies assumed that some of the deficits of BHK cells are due to a generally reduced capacity for virus attachment [12], Clarke and Spier proposed that the susceptibility of a cell population is determined by the proportion of susceptible and resistant cells within the population [9].

However, the mechanisms that determine susceptibility are still not fully solved. This study investigates why FMDV serotype Asia-1 in particular is disadvantaged in infecting BHK cells in the context of the underlying mechanisms that form the basis of cell susceptibility to infection with FMDV.

## 2. Materials and Methods

### 2.1. Cells

The suspension cell line BHK21C13-2P (BHK-2P) provided by the European Collection of Authenticated Cell Cultures (ECACC 84111301), was adapted to grow in the serum-free Cellvento^TM^ BHK200 medium (Merck KGaA, Darmstadt, Germany) in TubeSpin^®^ bioreactors (TPP Techno Plastic Products AG, Trasadingen, Switzerland), cultured in a shaker incubator with 320 revolutions per minute (rpm) at 37 °C, 5% carbon dioxide and 80% relative humidity.

An adherent BHK21 cell line (clone 13, derived from American Type Culture Collection [ATCC] CCL-10™, held as CCLV-RIE 179 in the Collection of Cell Lines in Veterinary Medicine, Friedrich-Loeffler-Institut, Greifswald, Germany; in short: BHK179) served as a positive control for virus susceptibility. It was cultured in Minimum Essential Medium Eagle (MEM) supplemented with Hanks’ and Earle’s salts (Sigma-Aldrich, St. Louis, MO, USA) with 10% fetal bovine serum (FBS) during maintenance and passaging, and with 5% FBS during infection experiments. Cells were incubated in sealed flasks at 37 °C.

The assessment of cell death and viability is fundamentally different between adherent and suspension cells. In adherent cells, cell death can be visually evaluated and is reported as the percentage of cells in a field of view that exhibit cytopathic effect (CPE). Cell death in suspension culture cannot as easily be evaluated under a microscope. Cell viability is quantified by a dye exclusion method. In addition, to account for the rapid growth of healthy suspension cells, the cell density in an infected culture is compared to an identically seeded negative culture. CPE and cell viability are related concepts, but are not identical.

The Chinese hamster ovary (CHO) cell lines CHO-K1 (ATCC CCL-61, held as CCLV-RIE 134) and CHO677 (CRL 2244, held as CCLV-RIE 1524) were maintained in Ham’s MEM mixed 1:2 with Iscove’s Modified Dulbecco’s Medium (Thermo Fisher Scientific, Darmstadt, Germany) and with 10% FBS at 37 °C in sealed flasks.

### 2.2. Susceptibility of BHK-2P for FMDV Stock Viruses

Different Asia-1 isolates (Asia TUR 6/2014, Asia TUR 6/2014-PT, Asia HKN 5/2005, Asia PAK 5/2012) were selected from archival stocks of the Friedrich-Loeffler-Institut (FLI) and were tested for their ability to successfully infect BHK-2P cells. Virus was first grown on adherent BHK164 cells and the gained supernatant was used to infect the BHK-2P, followed by repeated passaging in BHK-2P. Successful virus replication was defined as a decrease in cell viability and growth. Cell viability was determined using trypan blue (Bio-Rad, Hercules, CA, USA) and an automatic cell counter (Bio-Rad, model TC20™, Munich, Germany). To check the supernatant for remaining infectivity, it was passaged on adherent cells (infection control).

For further experiments, a representative Asia-1 strain, the Shamir/ISR/1989 isolate, was used. As comparison, a second strain, FMDV A_24_ Cruzeiro, which can infect the adherent and the suspension cell lines, was used in selected experiments. A third strain, FMDV O_1_ Manisa, was used in experiments testing the pH sensitivity. All virus strains were used as cell culture supernatant. For a detailed listing of the used isolates and passage histories see the Supplementary Materials (Table S1).

### 2.3. Virus Titrations

Viral titers were estimated by endpoint titration. Titers are expressed as 50% tissue culture infectious doses (TCID_50_) per 100 µL calculated with the Spearman-Kärber method [13,14]. Titrating BHK179-passaged virus on BHK179 cells could bias the results, therefore all titrations were performed on adherent BHK21 clone “Tübingen” cells (CCLV-RIE 164; referred to as BHK164).

### 2.4. Storage Conditions

To rule out that the difficulties of Asia-1 Shamir to infect BHK-2P are due to the media in which the cells are maintained, different storage conditions were tested. Equal amounts of virus (Asia-1 Shamir or A_24_ Cruzeiro) were added in a final dilution of 1:100 to 1.5 mL of serum-free Cellvento^TM^ BHK200, Cellvento^TM^ BHK200 with 5% FBS, or MEM with 5% FBS. The samples were incubated at 37 °C with agitation (350 rpm) for 0, 4, 8, 12, or 24 h and the remaining infectivity was determined as described above. All experiments were independently performed three times.

### 2.5. pH Sensitivity

A protocol already published by Martín-Acebes et al. [15] was used with some modifications. In short, equal amounts of virus (Asia-1 Shamir, O_1_ Manisa or A_24_ Cruzeiro) were mixed with 1 mL phosphate-buffered saline (PBS) solutions of different pH (7.5, 7.0, 6.8, 6.5, 5.5) in a final dilution of 1:100 and incubated for 30 min at room temperature. Afterwards, the samples were neutralized with 1M Tris-HCl (pH 8.0). The remaining infectivity in each sample was determined by titration as described above. All experiments were independently performed three times.

### 2.6. Blockage of Endosomal Acidification with Ammonium Chloride

The assay was performed as published [15] with some modifications. BHK179 monolayers as well as aliquots of BHK-2P suspension cells were treated with culture medium supplemented with 25 mM or 50 mM NH_4_Cl (Sigma-Aldrich) one hour prior to infection. The same concentration of NH_4_Cl was maintained throughout the rest of the infection. Cells in culture medium without supplementation served as positive controls. Adherent cells were infected with Asia-1 Shamir or A_24_ Cruzeiro, whereas suspension cells were only infected with A_24_ Cruzeiro adapted to BHK-2P. The cells were incubated for six hours post infection and virus was harvested by freezing at −70 °C and thawing. Viral yields were estimated by titration as described above. All experiments were performed independently in duplicates three times.

### 2.7. RNA Transfection and Immunofluorescence

#### 2.7.1. RNA Transfection

Aliquots of Asia-1 and A_24_ Cruzeiro grown in BHK179 were used as source of FMDV RNA. Nucleic acid was isolated manually with the QIAamp^®^ Viral RNA Mini Kit (Qiagen GmbH, Hilden, Germany) as described by the manufacturer. The RNA content of the eluates was quantified with a NanoDrop™ 2000c spectrophotometer (Thermo Fisher Scientific, Wilmington, DE, USA), and FMDV genome load was assessed using a real-time reverse-transcription polymerase chain reaction (RT-PCR) assay targeting the 3D-coding region of the viral genome [16].

BHK-2P cells grown in suspension were transfected with the RNA using Lipofectamine 3000 (Invitrogen, Carlsbad, CA, USA) according to the manufacturer’s instructions. In short, 10 µL of RNA and 0.75 µL or 1.5 µL of Lipofectamine diluted in Cellvento™ BHK200 were mixed and incubated at room temperature for 20 min to allow complex formation. Afterwards, these mixtures were transferred to 78 µL of BHK-2P cells (approximately 1 × 10^5^ cells/mL) in 24-well plates and medium was added up to 200 µL. The cells were incubated for 4 h at 37 °C and 700 µL medium was then added, followed by further incubation at 37 °C for 24 h. After 24 h, the entire contents of the wells were harvested and frozen at −70 °C. The thawed cell lysate was clarified by centrifugation and used to treat adherent BHK164 cells.

#### 2.7.2. Immunofluorescent Staining

Confluent monolayers of BHK164 cells grown in 96-well plates were treated with 50 µL of clarified cell lysate harvested directly from the transfected cells (see Section 2.7.1), as well as with stock virus (positive control) and with 10 µL of the extracted RNA itself (negative control). Cells were then incubated at 37 °C for 4h. After the incubation period, the supernatant was discarded, cells were washed with PBS once and fixed with 4% (*w*/*v*) paraformaldehyde (Sigma) dissolved in PBS for 20 min at room temperature (RT). Cells were washed again once with PBS and permeabilized with PBS with 0.1% (*v*/*v*) Triton X-100 for 5 min at RT. The permeabilized cells were then incubated with the primary antibody (polyclonal serum from cattle experimentally infected with O_1_ Manisa) diluted 1:200 in PBS with 0.05% (*v*/*v*) polysorbate 20 (Tween; in short: PBS/T), for 20 minutes at RT. Next, cells were washed two times with PBS/T and incubated with the secondary antibody, rabbit anti-bovine immunoglobulin G (IgG) conjugated with fluorescein (FITC) (Thermo Fisher Scientific, Darmstadt, Germany) (diluted 1:500 in PBS/T) for 20 min at RT. Afterwards, cells were washed two times with PBS/T, nuclei were counterstained with DAPI (4′,6-diamidino-2′-phenylindole, dihydrochloride, Thermo Fisher Scientific; diluted 1:1000 in PBS/T) for 5 min at RT, followed by three washes with PBS/T. Cells were examined with a fluorescence microscope (Axio Vert.A1; Carl Zeiss, Oberkochen, Germany) and data were collected utilizing the prepared controls of the anti-FMDV antibodies with uninfected cells to give the negative background levels. The captured images were processed with ImageJ to globally adjust contrast and brightness and merge the green and blue color channels [17].

### 2.8. Susceptibility of Other Cell Lines and Cross-Infection Experiments

#### 2.8.1. Susceptibility of Other Cell Lines

To determine if FMDV Asia-1 Shamir generally cannot infect suspension cells or if that failure is more specifically caused by changes in each cell line during the adaption to suspension culture, several other lines of BHK suspension cells were inoculated with the virus: The adherent original BHK21, clone 13 (BHK21C13; derived from ATCC CCL-10™), maintained in GMEM with 8.5% FBS (referred to as cell line #1), the same cells adapted to grow in suspension by sequential withdrawal of serum (#3), the previously introduced BHK-2P (Section 2.1) maintained either in GMEM with 10% FBS (#2) or adapted to BHK200 in two different processes (lines #4 and #5), plus four additional suspension cell lines, all maintained in BHK200 (BHK21-C, #6; BHK21-Hektor, #7; BHK21-InVitrus, #8 (Sigma); production BHK, #9). All cell lines except #8 were provided by Merck KGaA, Darmstadt, Germany. Successful virus replication in suspension cells was defined as a decrease in cell viability in comparison to the uninfected cells (negative control, NC), together with a decrease in the required infection volume. In adherent cell lines, the occurrence of cytopathic effect (CPE) was defined as successful virus replication.

#### 2.8.2. Infection and Virus Adaption Experiments

All susceptible cell lines were used to repeatedly passage Asia-1 Shamir. The virus grown in these cell lines was then put back on BHK-2P to determine if it could now infect them.

Infection experiments and adaption studies were performed with the following virus isolates, obtained by passaging Asia-1 Shamir in the cell lines described in the previous section: #3 Asia-1, after four passages on BHK21C13; #8 Asia-1, after seven passages on BHK21-InVitrus and #9 Asia-1, after five passages on production BHK (see also Table S1). BHK-2P cells were seeded with a cell density of 1 × 10^6^ cells/mL and infected with 5 mL cell culture supernatant, corresponding to 1/6 of the total culture volume of 30 mL. After an incubation period of 20–24 h, cell viability was determined. Once the cell viability dropped below 15%, the infection volume for the next passage was reduced. The adaption of the virus was considered successful when an infection volume of 5% of the total culture volume (i.e., 1.5 mL) was sufficient to kill at least 90% of the cells after 20–24 h. To prove successful virus replication, one aliquot taken at the time of infection (0 h post infection, hpi) and one aliquot taken at the end of the incubation period (24 hpi) were titrated as described above (Section 2.3). A mock-infected negative control was passaged under the same conditions as the virus.

#### 2.8.3. Attachment Test

BHK179 cell monolayers were washed with medium and then incubated with Asia-1 Shamir (10^6.1^ TCID_50_), Asia-#3 (10^5.1^ TCID_50_), Asia-#8 (10^5.5^ TCID_50_), Asia-#9 (10^4.7^ TCID_50_) or A_24_ Cruzeiro (10^5.4^ TCID_50_) in 1 mL of MEM with 5% FBS. BHK-2P were adjusted to 1 × 10^6^ cells per mL, centrifuged at 300× *g* for 5 min and resuspended in 1 mL fresh Cellvento^TM^ BHK200 with the same viruses and doses as used for the adherent cells.

The cells were kept on ice to prevent the internalization of the virus [18]. After 15 min, the supernatant was discarded and the cells were washed two times with medium to remove unbound virus. Monolayers were detached with 0.05% trypsin and 0.02% ethylenediaminetetraacetic acid (EDTA). The cell pellet was collected in 1 mL MEM with 5% FBS and titrated. All experiments were performed in duplicate and repeated for a total of three times.

#### 2.8.4. Sequence and Structure Analysis

FMDV RNA was extracted from the original virus stock of Asia-1 Shamir and the final passages of #3 Asia-1, #8 Asia-1 and #9 Asia-1 using TRIzol^®^ LS Reagent (Invitrogen, Karlsruhe, Germany) and the RNeasy^®^ Mini Kit (Qiagen, Hilden, Germany) according to the manufacturers’ instructions.

Reverse transcription and PCR was done using a method previously described [19]. Three additional primer pairs were used to fill in gaps (VP1-3165F, VP1-3632R, VP3-2835F, VP3-3217R, 3D-7320F and 3D-8097R, see Table S3). The nucleotide sequences were assembled and mapped with Geneious (Biomatters Limited, Auckland, New Zealand) against the complete published sequence for Asia-1 Shamir (Genbank accession no. JF739177). Sequences of the initial Asia-1 Shamir strain and the #3-, #8- and #9-Asia-1 isolates have been uploaded to Genbank (MF063053-MF063056).

The capsid map was created with the Virus Particle Explorer (VIPER, http://viperdb.scripps.edu/) [20] using FMDV O1/BFS/1860 and A10/Argentina/61 as templates (Protein Data Bank accessions 1BBT [21] and 1ZBE [22]). The crystallographic structure of the mutations located in the capsid pentamer was analyzed with the UCSF Chimera package [23], using 1ZBE as template. Chimera is developed by the Resource for Biocomputing, Visualization, and Informatics at the University of California, San Francisco, CA, USA (supported by NIGMS P41-GM103311).

### 2.9. Infectivity Assay on Receptor-Deficient Cells

Infectivity assays on CHO K1 and CHO677 cells were performed as described by Jackson et al. [24] with one modification: harvested virus-infected CHO cells were titrated on BHK164. All experiments were performed in duplicate and repeated for a total of three times.

### 2.10. Virus Neutralization Test

The virus neutralization test (VNT) was performed with Asia-1 Shamir, Asia-#3, -#8 and -#9 and BHK164 cells as prescribed by the World Organisation for Animal Health (OIE) [25]. Neutralization titers are expressed as the log_10_ of the reciprocal of the final dilution of serum where 50% of wells are protected, i.e., show no CPE. Two different sera of bovine origin were used for the VNT. Serum “P2/99” had been collected 21 days after vaccination (dpv) with a commercial Asia-1 vaccine (Bayer AG, lot W4829). Serum “RD460” was taken 19 days after infection with Asia-1 stock virus (second passage on BHK164). The experiments were conducted in duplicates, three times independently. R_1_ values were calculated by dividing the mean neutralization titer of each serum against the adapted virus by the mean neutralization titer of the serum against the original isolate.

### 2.11. Statistical Analysis

In all experiments, the differences between treatment groups were evaluated with linear mixed-effects models using R (http://www.r-project.org) and lme4 [26]. Wald chi-square tests for fixed effects and their interactions were calculated with the car and phia packages. *p*-Values of <0.01 were considered significant.

## 3. Results

### 3.1. FMDV Serotype Asia-1 Cannot Infect BHK-2P Cells

Three independent attempts have been performed to infect the suspension cell line BHK-2P with the Asia-1 Shamir isolate as well as single attempts with other Asia-1 isolates. Supernatant to inoculate the first passage on BHK-2P was obtained from adherent BHK cells that also served as positive controls for successful virus replication. After each passage, BHK-2P were lysed by freezing and thawing, and the clarified lysate was used to inoculate a fresh culture of BHK-2P cells. After repeated passaging without any decrease in cell viability, lysate of the last BHK-2P passage was transferred to adherent BHK or LFBKαvβ6 to check for residual infectivity (infection control). No live virus was detected in any of these experiments (see Table S4).

### 3.2. The Ability of FMDV to Infect BHK-2P is not Dependent on the Culture Environment

Different culture conditions such as the media in which cells and virus are cultured as well as its pH were examined for their influence on virus particle stability or cell susceptibility.

First, equal amounts of FMDV A_24_ Cruzeiro and Asia-1 were incubated over a period of 24 h in three different culture media. Every four hours, one aliquot was titrated to determine the remaining infectivity. Because BHK-2P are maintained in media without serum or any other animal-derived components, the experiments were designed to investigate whether the medium alone has detrimental effects on virus particle stability.

**Figure 1:**
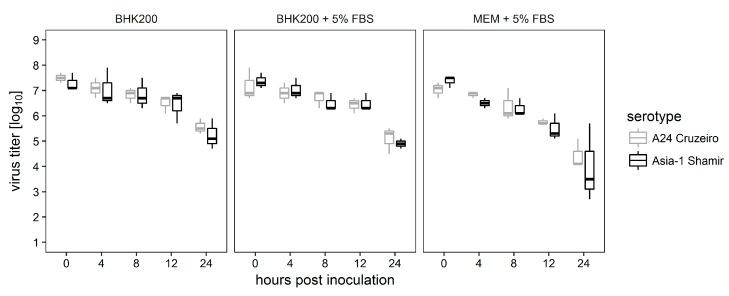
Storage of FMDV A_24_ Cruzeiro and Asia-1 Shamir in different culture media for 24 h. Equal amounts of virus were incubated in serum-free Cellvento™ BHK200 media, Cellvento™ BHK200 supplemented with 5% fetal bovine serum (FBS), and Minimum Essential Medium Eagle (MEM) supplemented with 5% FBS. Every four hours, one aliquot was titrated to determine remaining infectivity. All experiments were independently performed three times.

Viral titers declined over time for both serotypes. Statistical analysis did not show any significant differences between A_24_ Cruzeiro and Asia-1 Shamir in any of the three media (Figure 1). Therefore, the media does not directly influence the potential of the virus to infect the BHK-2P cells.

**Figure 2:**
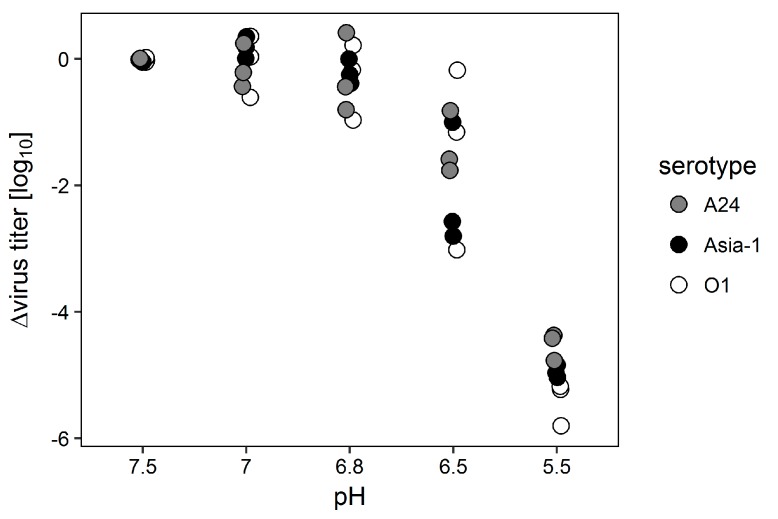
Incubation of FMDV A_24_ Cruzeiro, O_1_ Manisa and Asia-1 Shamir in PBS solutions of different pH for 30 min. Equal amounts of virus were incubated at pH 7.5, 7.0, 6.8, 6.5 and 5.5 for 30 min. The solutions were neutralized and remaining infectivity was determined by titration. Values on the y-axis represent the reduction in titer compared to virus incubated at pH 7.5. All experiments were independently performed three times.

Next, the pH-dependent disintegration of the serotype Asia-1 isolate was compared to serotypes A or O. Equal amounts of each virus were incubated in PBS solutions of different pH for 30 min and remaining infectivity was determined by titration. Results indicate a wide spread in infectivity for the solution with pH 6.5 but overall show no difference between serotypes for the different pH conditions (Figure 2).

**Figure 3:**
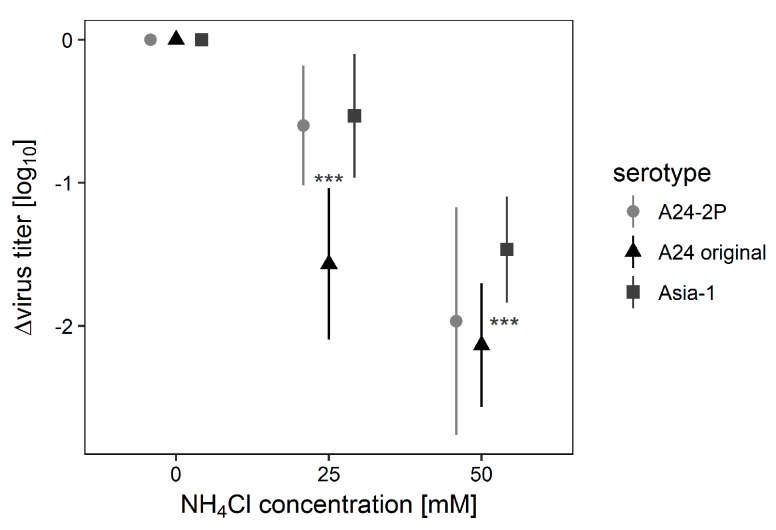
Different NH_4_Cl sensitivity of FMDV serotypes A and Asia-1. BHK monolayers as well as suspension cells were treated with 50 mM or 25 mM ammonium chloride or left untreated. BHK179 were infected with FMDV Asia-1 Shamir and A_24_ Cruzeiro (original), and BHK-2P were infected with the adapted virus strain A24-2P. After 24 h of incubation, the remaining infectivity was determined by titration. Values on the y-axis represent the reduction in virus yield compared to untreated cells. Experiments were performed in duplicates three times independently. Significance code: *** *p* < 0.001.

Finally, BHK179 cell monolayers as well as BHK-2P suspension cells were treated with 25 mM or 50 mM ammonium chloride. Untreated cells served as controls. Virus yields for all strains were decreased by the ammonium chloride treatment. The original A_24_ virus strain in BHK179 cells was significantly more affected by the ammonium chloride treatment than the other strains (Figure 3), but there was no significant difference between Asia-1 in adherent BHK179 and the 2P-adapted strain of A_24_ in suspension culture. Therefore, even though adaption to suspension cells increased the ammonium chloride resistance of A_24_, Asia-1 Shamir does not display a degree of susceptibility that could explain its inability to infect BHK-2P cells.

In summary, environmental conditions such as cell culture media and pH, as well as endosome acidification, do not explain the inability of FMDV Asia-1 Shamir to infect certain cell lines.

### 3.3. BHK-2P Cells Can Produce Infectious Asia-1 FMDV

**Figure 4:**
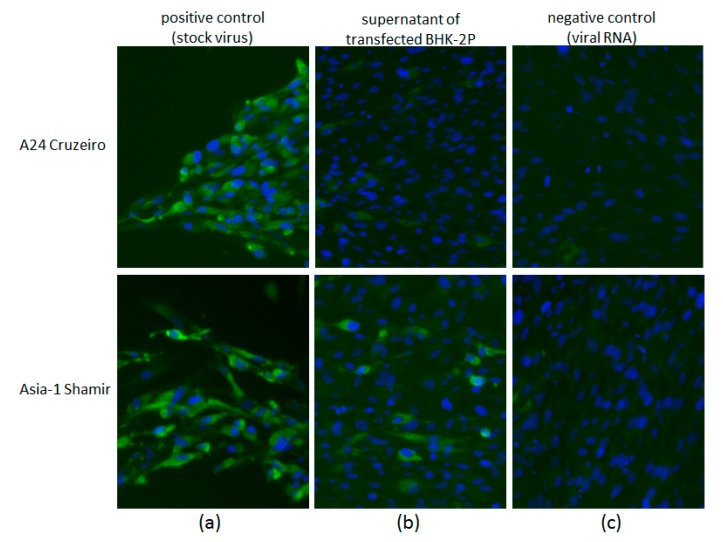
Detection of FMDV antigen in adherent BHK164 after treatment with supernatant from transfected BHK-2P. Adherent BHK164 were incubated with stock virus, clarified supernatant from transfected BHK-2P, or viral RNA for 4 h at 37 °C, then fixed and stained with serum from FMDV-infected cattle (green staining). Cell nuclei are shown in blue (**a**) Cells infected with A_24_ Cruzeiro and Asia-1 Shamir stock virus as positive controls (PC); (**b**) Cells incubated with supernatant from transfected BHK-2P; (**c**) Cells incubated with viral RNA alone (no transfection reagent) as negative controls (NC).

Viral RNA of A_24_ Cruzeiro and Asia-1 Shamir was extracted und transfected into BHK-2P. When the supernatant of the transfected cells was added to BHK164 monolayers, they showed strong CPE and stained positive for FMDV antigen after 24 h of incubation (Figure 4). However, virus production in the BHK-2P cells occurred only in a single cycle, i.e., while the transfected cells did produce virus, the virus that was released was not amplified by a passage in BHK-2P cells. These results indicate that the reduced susceptibility of BHK-2P cells is related to a blocked virus entry or an inefficient virus adhesion at the cell surface, but not caused by a defect in the replication of the virus and the production of infectious progeny. The cells are resistant but permissive.

### 3.4. Cellular Resistance Can be Overcome by Virus Adaption to a “Wet Nurse” Cell Line

Several different suspension cell lines as well as the parental adherent line were inoculated with Asia-1 Shamir to test their susceptibility for this virus. Results show that the BHK-2P line was not susceptible to the original Asia-1 Shamir isolate under any culture conditions (#2, #4, #5). Two of the other suspension cell lines tested (#6, #7) also did not support the replication of Asia-1 Shamir.

Three cell lines proved susceptible to FMDV Asia-1 Shamir: an adherent BHK21 clone 13 adapted to grow in suspension in serum-free medium after sequential withdrawal of serum (short: BHK21C13, “#3”), the suspension cell line BHK21-InVitrus (“#8”), and another production BHK-derived suspension cell line (short: production BHK, “#9”) (Table S2).

After several passages on these susceptible cells, the recovered Asia-1 viruses were tested for their capacity to infect BHK-2P cells. With these viruses, productive infection in BHK-2P could be achieved after serial passaging (Table 1). Adaptation was considered complete when inoculation of 0.5 mL of supernatant from the previous passage led to a 90% decrease in cell viability within 20 h. The fully BHK-2P-adapted virus isolates (#3 Asia-1, #8 Asia-1 and #9 Asia-1) were then sequenced and compared with the genome of the original Asia-1 Shamir virus strain (Table S6). The quickest adaption onto BHK-2P was achieved for #9 Asia-1 after only four passages. The virus strains #3 Asia-1 and #8 Asia-1 required nearly twice as many passages for complete adaption as #9 Asia-1.

**Figure 5:**
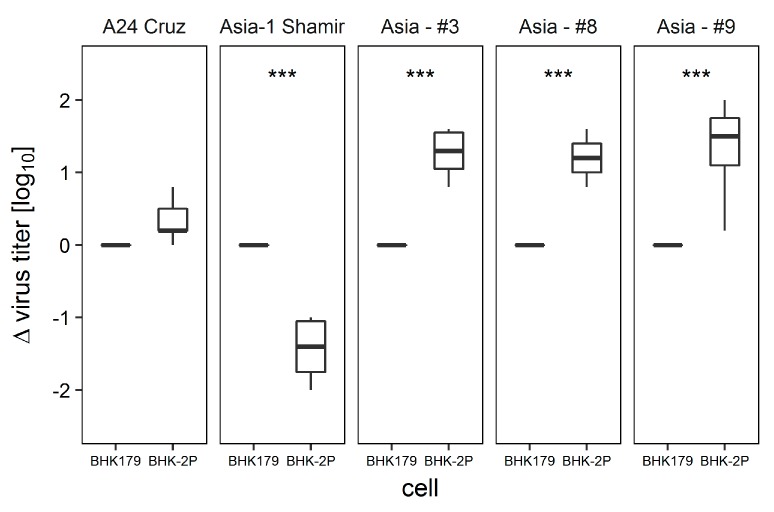
Infectivity after attachment of A_24_ Cruzeiro, Asia-1 Shamir and adapted Asia-1 isolates to BHK179 or BHK-2P. The viruses were incubated either with the adherent cell line BHK179 or the suspension cell line BHK-2P for 15 min on ice to prevent internalization. Cells were washed to remove unbound virus and titrated. The *y*-axis shows the difference in titer between the BHK-2P and the BHK179 cell preparation. Experiments were performed in duplicates and repeated for a total of three times. Significance code: *** *p* < 0.001.

FMDV A_24_ Cruzeiro, Asia-1 Shamir and the adapted Asia-1 isolates #3, #8 and #9 were incubated with adherent BHK179 cells or BHK-2P for 15 min. Cells and virus were incubated on ice to avoid virus uptake into the cells. Afterwards, the cells were thoroughly washed and the amount of bound virus was determined by titration. The comparison between the two cell lines revealed no significant difference for A_24_ Cruzeiro (log_10_ titer_179_ with standard deviation = 4.6 ± 0.2; titer_-2P_ = 4.9 ± 0.3) but a highly significant loss of titer for Asia-1 Shamir (titer_179_ = 4.5 ± 0.6; titer_-2P_ = 3.1 ± 0.5). In contrast, Asia-1 isolates #3, #8 and #9 showed a significantly higher titer on BHK-2P (log_10_ titers = 5.5 ± 0.3; 5.7 ± 0.3; 4.3 ± 0.3, respectively) in comparison to the adherent BHK179 (log_10_ titers = 4.3 ± 0.2; 4.5 ± 0.4; 3.0 ± 0.5, respectively) (Figure 5).

### 3.5. Sequence Analysis Revealed Mutations in the Five-Fold Axis and Extended Receptor Tropism

**Figure 6:**
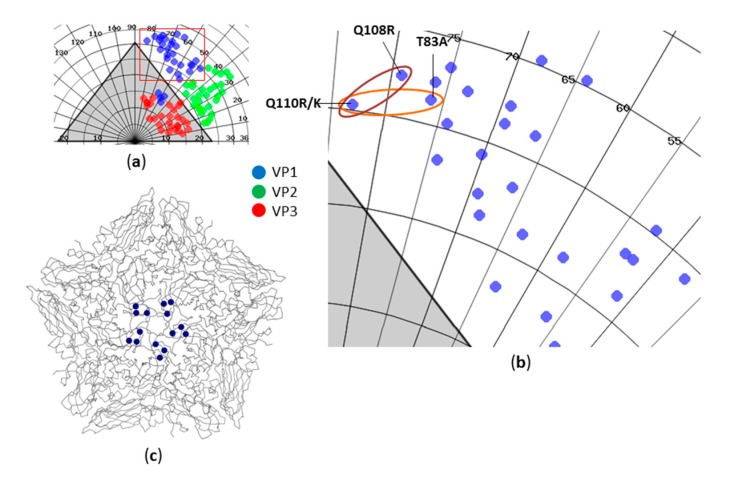
Capsid maps of the outer surface molecules of FMDV. The capsid map was created with VIPER [20] using FMDV O_1_/BFS/1860 and A_10_/Argentina/61 as templates: (**a**) overview of the exposed residues on the outside of the virus capsid, color-coded for VP1 (blue), VP2 (green) and VP3 (red); (**b**) zoom on the location where mutations were found in #3 Asia-1, #8 Asia-1 and #9 Asia-1; (**c**) crystallographic structure of an FMDV capsid pentamer. The VP1 residues 83, 109 and 110 are highlighted. All three mutations are in close spatial proximity to each other and to the fivefold symmetry axis. Residue 110 was mutated in all three viruses, and it appears that this exchange was only possible in combination with a second exchange: either Q108R (in #3 Asia-1 and #8 Asia-1) or T83A (in #9 Asia-1).

For #9 Asia-1, five non-synonymous mutations (i.e., amino acid changes) were found in the genome of the fully adapted virus. The virus strains #3 Asia-1 and #8 Asia-1 acquired three identical non-synonymous mutations during the adaption process. All three virus strains replaced an uncharged glutamine (Q) at residue 110 of the VP1 capsid protein with either a positively charged arginine (R) (#3 Asia-1 and #8 Asia-1) or a positively charged lysine (K) (#9 Asia-1). This mutation always occurred together with a second exchange in VP1, either Q108R for #3 Asia-1 and #8 Asia-1 or T83A (threonine to alanine) for #9 Asia-1. All modified amino acids are exposed on the outer capsid surface and are located in close spatial proximity to each other with no known interactions at any protomer interfaces (Figure 6).

Besides that, virus strain #9 Asia-1 exhibited one additional mutation in the C-terminus of VP1 (E202K) and an amino acid exchange from a negatively charged glutamic acid to a positively charged lysine at position 59 (E59K) of the VP3 protein (Table 2). The mutations in the C-terminus of VP1 as well as the mutation in the VP3 protein are part of the heparin-binding site, which is formed by residues 55–58 of VP3 and the following residues 58–60 of the successional loop, the C- terminus of VP1 (residues 195–197) and residues 133–138 of the VP2 protein [22].

Moreover, all three virus strains acquired the same heterologous mutation in the non-structural 2C protein (K285Q), which is responsible for RNA replication [27].

To determine if the Asia-1 mutants adapted to use heparan sulfate (HS) or a non-integrin, non-HS receptor, the original virus isolate Asia-1 Shamir and the adapted mutants were incubated on different CHO cell lines. CHO-K1 cells express HS but none of the integrins αvβ1, αvβ3, αvβ6 or αvβ8. CHO677 cells are also deficient for these integrins, but do not express HS. All three adapted isolates were able to grow on both cell lines, while the wildtype Asia-1 Shamir was unable to infect either of them (Table 3). For growth on CHO-K1 cells, no significant differences between #3 Asia-1, #8 Asia-1 and #9 Asia-1 could be found. Interestingly, for the #9 Asia-1 isolate, there was also no significant difference between CHO-K1 or CHO677 cells, whereas titers of #3 Asia-1 and #8 Asia-1 were significantly reduced on CHO677 in comparison to CHO-K1 (*p* < 0.001). These findings suggest that all three adapted isolates extended their receptor usage and that the additional mutations in the capsid of #9 Asia-1 offer an advantage in cell culture infection compared to #3 and #8 Asia-1.

### 3.6. Virus Adaptation Alters Neutralization Profile

**Figure 7:**
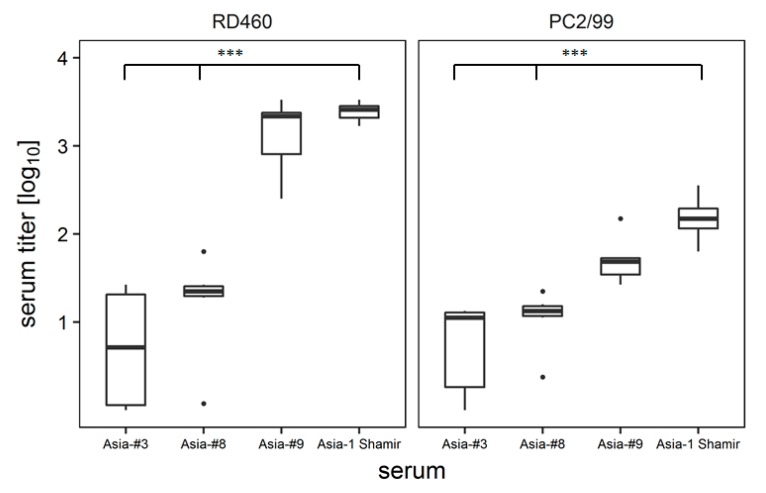
Serum neutralization tests revealed an altered neutralization profile of the adapted viruses. Asia-1 Shamir and the adapted Asia-1 isolates #3, #8 and #9 were incubated with serum from experimentally infected (“RD460”) or vaccinated (“PC2/99”) cattle. Experiments were performed in duplicates and repeated for a total of three times. Significance code: *** *p* < 0.001.

Virus neutralization tests were conducted to determine if any antigenic changes took place during adaptation of Asia-1 Shamir to the BHK-2P cell line. Serum of bovine origin, collected after infection with Asia-1 Shamir (“RD460”), strongly neutralized the original Asia-1 and the adapted #9 isolate (r_1_ value = 0.92), but showed a highly significant loss in titer against the #3 and #8 adapted Asia-1 isolates (r_1_ = 0.21 and 0.36, respectively). The neutralization titers of a second serum, taken from a vaccinated animal (“PC2/99”), generally were lower compared to the serum of the infected animal. Similar to the infected animal, the loss in neutralizing activity of this serum for the adapted isolates #3 and #8 (r_1_ = 0.33 and 0.48) was highly significant. The titers obtained with the adapted isolate #9 (r_1_ = 0.78) were reduced compared to the original Asia-1 isolate, but the difference was not significant (*p* = 0.05) (Figure 7).

## 4. Discussion

Since more than fifty years ago, BHK cells are the cell line of choice for propagation of FMDV and production of FMDV vaccines [2], even though specific problems with this cell line have been known for at least 35 years [9]. In addition to alterations in cell morphology and reduced surface expression of integrins, especially in BHK suspension cells [6], the susceptibility of BHK cells for FMDV infection is also known to change [9]. While some BHK cell lines lose the ability for efficient FMDV production on repeated subculturing, other BHK cells do not seem to be susceptible to FMDV Asia-1 at all. In this context, strains of serotype Asia-1 seem to be the most sensitive [9]. Previous investigations of this topic have suggested two possible reasons for the resistance of BHK cells. Diderholme and Dinter proposed resistance due to deficiencies in virus attachment at the cell surface [12], while Clarke and Spier assumed that cell populations are heterogeneous and the overall susceptibility of a cell line depends on the relative proportions of cells with different susceptibilities [9]. Inhibition of viral replication can occur at any point of the viral growth cycle [10] and the exact underlying mechanisms remain unclear.

With serotype Asia-1 being an indicator for the general FMDV susceptibility of a cell line, Asia-1 Shamir was chosen for our investigations. At first, the study focused on the exclusion of external factors in the culture environment that might influence virus particle stability in a serotype-specific manner and thus bias the results. Serum-free media often have reduced pH buffering capacity and fewer stabilizing components compared to serum-containing media [28]. In industrial production, the pH is regulated automatically, but in our small-scale experimental setting no such automatic control was possible. Within the scope of our experiments, the virus particle stability was not dependent on the culture medium or pH and did not differ between serotypes. Similarly, the sensitivity towards ammonium chloride was the same for Asia-1 Shamir, which was not able to infect BHK-2P cells, and for the 2P-adapted A_24_ Cruzeiro (A24-2P) virus strain. Based on this, external factors that might influence the experimental setting and can inhibit infection right from the start due to particle instability were excluded.

Clarke and Spier have argued that the resistance of a cell line to virus replication is not due to an inhibited release of newly synthesized virus particles [10]. This was confirmed by the results of the present study. Transfection experiments with viral RNA showed a single cycle of virus replication after transfection of BHK-2P cells with viral RNA of Asia-1 Shamir. The production of stable and infectious virus particles was confirmed by passaging the supernatant of the transfected cell culture on to adherent BHK cells. This leads to the conclusion that BHK-2P are indeed capable of virus production, and provides further evidence that their resistance to infection with FMDV Asia-1 is caused by a deficiency in cell binding, entry or uncoating of the virus particle.

Our experiments revealed three susceptible BHK suspension cultures (BHK21C13, BHK21-InVitrus, production BHK). Once Asia-1 Shamir had been successfully adapted to these three cell lines, the adapted strains were used in another attempt to infect BHK-2P cells. Surprisingly, all three pre-adapted strains replicated in BHK-2P and good viral growth was achieved after a few passages.

A previous study proposed a restriction at the stage of virus entry and/or uncoating as reason for the cellular resistance to FMDV, while a lack of virus attachment to the cell surface had been ruled out [10]. Therefore, the next investigations focused on the cell-virus interactions. The attachment capacity of BHK-2P for Asia-1 Shamir was significantly reduced in comparison to BHK179. On the other hand, the BHK-2P adapted Asia-1 isolates attached significantly stronger to BHK-2P than BHK179. These results provide an indication that BHK-2P cells have an altered surface structure in comparison to BHK179 and that the natural isolates of FMDV serotype Asia-1 are disadvantaged in utilizing the presented surface molecules for attachment and entry. FMDV is able to utilize four different integrin molecules for cell binding: αvβ1, αvβ3, αvβ6 and αvβ8 [24,29,30,31]. The utilization of these receptors varies between serotypes, for example serotype O prefers integrin αvβ6, followed by αvβ1, and serotype A prefers integrin αvβ3 and αvβ6 [32]. For Asia-1 no such preferences are known. BHK cells only possess one of the four integrins, integrin αvβ1, which is even downregulated in suspension cells or highly passaged adherent cells [7]. Another possibility for FMDV binding is the utilization of heparan sulfate proteoglycan (HSPG) as a receptor, typically as an adaption to cell culture [33]. Depending on the receptor used, the virus is either internalized via caveola-mediated endocytosis (heparan sulfate) [34] or in a clathrin-dependent manner (integrins) [35]. Many structural changes take place when cells adapt to grow in suspension, amongst others a restructuring of the cytoskeleton with disappearance of actin stress fibers [7]. For CHO cells, changes in the expression and clustering of integrins have been described after adaption to grow in suspension [36].

Sequencing of the viral genome revealed three amino acid exchanges (VP1: Q108R, Q110R; 2C: K285Q) which were shared by two (#3 Asia-1, #8 Asia-1) of the three isolates. The third isolate (#9 Asia-1) harbored the same mutation in the 2C protein and a similar mutation at position 110 of the VP1 protein (Q to K). Residue 108 was conserved in #9 Asia-1, but there was another amino acid exchange (T83A) in close spatial proximity to the mutations acquired by the other two isolates. In addition, the genome of the third isolate carried two more mutations, introducing positive charges to regions of VP1 (E202K) and VP3 (E59K) that form the HSPG-binding pocket in type O and A viruses [22,37]. The accumulation of positive charges on the capsid surface argues for the acquisition of heparan sulfate binding, particularly for #9 Asia-1. The mutations at residues 108 and 110 of VP1, however, fall outside of the canonical HSPG binding pocket. HSPG binds to the center of the protomer [38], whereas residues 108 and 110 are located at the edge of the protomer, next to the five-fold symmetry axis of the capsid pentamer. The acquisition of positively charged amino acids in this region is associated with the binding of a third, not conclusively identified FMDV receptor [37,38]. In fact, the introduction of a positive charge at residue 110 of VP1 into an FMDV Asia-1 strain enabled it to infect αvβ6- and HSPG-deficient CHO cells, provided that a second positive charge existed in close proximity—residue 109 as described by Berryman et al. [38]. In the present study, all three isolates gained the ability to successfully infect CHO-K1 cells as well as HSPG-deficient CHO677 cells. Strikingly, #9 Asia-1 developed significantly higher titers when replicating in CHO677 cells in comparison to isolates #3 and #8. This raises the question whether the introduced HS-specific mutations also support the usage of another, unknown receptor.

It is also interesting that all three virus isolates exhibit the same mutation in the non-structural 2C protein. This protein is highly conserved among the different serotypes with >85% identity of the amino acids [39] and has important functions during virus replication [27]. The 2C protein is a membrane-binding component of the replication complex with additional functions, including RNA binding activities, ATPase and GTPase activities as well as the induction of apoptosis in BHK cells [39,40,41]. Recent studies revealed an interaction between 2C and cellular Beclin1, which blocks autophagosome fusion to lysosomes and prevents RNA degradation as well as enhances viral replication [39]. Possible binding sites were predicted at positions 225–280 and 288–294 within the 2C protein that consists of 318 amino acids in total [39]. The detected mutation within all three adapted Asia-1 strains is located at position 285, in between the possible binding sites for Beclin1. Another function of 2C is the interaction with cellular vimentin [42]. Vimentin is important for cell spreading in adherent cells [43], but culturing of cells in suspension leads to a decrease in vimentin biosynthesis [44]. During FMDV infection, vimentin builds cage-like structures around the non-structural 2C protein [42]. An intact vimentin network is indispensable for FMDV replication and its chemical disruption leads to a decrease in viral yield [42]. Although the observed mutation is distant from the predicted binding site for vimentin (at position 78–84), it is possible that the mutation in the 2C protein enhances viral growth within the cell, especially in the context of altered vimentin synthesis in BHK suspension cells.

Unfortunately, these studies did not fully elucidate the root cause of resistance of BHK-2P and other suspension cell lines towards native FMDV Asia-1. It appears that the infection can be impeded by the lack of suitable (or preferred) receptors on the cell surface. For instance, necessary receptors can be more accessible or available in higher quantity on the surface of susceptible cell lines than on resistant cell lines. By using a closely related but permissive cell line as a “wet nurse” for the adaption of the virus, this obstacle can be overcome. This is of high importance for the production of FMDV vaccine antigen, if the production cell line of choice shows resistance to some FMDV strains. Intriguingly, when incubating the adapted viruses with serum of infected or vaccinated animals, an altered neutralization profile compared to the original Asia-1 Shamir isolate became evident. This is an interesting finding, but does not prove that a vaccine made from these mutants would be without protective effect. BHK-2P cells do not seem to present receptor molecules on their surface for the virus to invade the cells via the “natural way”, i.e., by using integrins [45,46,47]. Therefore, alternative routes had to be found by the virus and were found in the mutations around the five-fold axis. At the same time, the structures important for natural infection, such as the RGD motif, remained unchanged. There is currently no indication that the induction of antibodies against these structures would be affected by the capsid mutations. Thus, infection or vaccination with the adapted viruses could still lead to an immune response that protects against infection with field isolates of Asia-1. Further studies, including animal trials, are necessary to answer these questions and to prove the quality and quantity of the antigen obtained with the described mutants and/or other strains that have been adapted in this rather circuitous manner.

## Figures and Tables

**Table 1:** Decrease of cell viability and the required infection volume during serial passaging of Asia-1 isolates with different original cell adaption background on BHK-2P.

**Passage No.**	**#3 Virus from BHK21C13 in Cellvento™ BHK200**					**NC**
	**Cell Viability 0 hpi**	**Cell Viability 20 hpi**	Viral Titer 0 hpi *	Viral Titer 20 hpi *	**Infection Volume**	**Cell Viability 20 hpi**
1	98%	63%	4.9	5.9	5 mL	99%
2	99%	73%	5.5	5.7	5 mL	98%
3	99%	79%	5.7	5.9	5 mL	96%
4	99%	60%	5.3	6.1	5 mL	99%
5	99%	13%	5.5	5.9	5 mL	98%
6	97%	6%	5.5	6.1	4 mL	97%
7	97%	16%	4.9	6.1	3 mL	99%
8	98%	5%	5.3	5.9	1.5 mL	97%
9	98%	8%	4.3	6.1	0.5 mL	96%
**Passage No.**	**#8 Virus from BHK21-InVitrus Cells in Cellvento™ BHK200**					**NC**
	**Cell Viability 0 hpi**	**Cell Viability 20 hpi**	Viral Titer 0 hpi *	VIRAL titer 20 hpi *	**Infection Volume**	**Cell Viability 20 hpi**
1	98%	60%	4.7	5.7	5mL	99%
2	99%	72%	5.5	5.9	5mL	98%
3	99%	86%	5.9	6.1	5 mL	96%
4	99%	53%	5.5	5.9	5 mL	99%
5	99%	16%	5.1	6.3	5 mL	98%
6	97%	10%	5.5	5.9	4 mL	97%
7	97%	12%	5.1	6.3	3 mL	99%
8	98%	6%	5.3	5.9	1.5 mL	97%
9	98%	9%	4.1	6.5	0.5 mL	96%
**Passage No.**	**#9 Virus from Production BHK Cells in Cellvento™ BHK200**					**NC**
	**Cell Viability 0 hpi**	**Cell Viability 20 hpi**	Viral Titer 0 hpi *	Viral Titer 20 hpi *	**Infection Volume**	**Cell Viability 20 hpi**
1	98%	61%	5.9	6.3	5 mL	99%
2	99%	8%	5.5	5.9	5mL	98%
3	99%	14%	5.1	6.1	3 mL	96%
4	99%	2%	4.9	6.1	1.5 mL	99%
5	99%	3%	3.9	5.7	0.5 mL	98%

* Titers are expressed in log_10_ TCID_50_ per 100 µL. 3.5 Attachment of FMDV to BHK-2P cells differs between virus isolates. hpi: Hours post-infection; NC: Negative control.

**Table 2:** Overview of amino acid changes during passaging of Asia-1 Shamir.

Protein	Structure *	Virus Isolate		
		#3 Asia-1	#8 Asia-1	#9 Asia-1
VP3	beta-B ‘knob’	-	-	E59K
VP1	capsid surface	-	-	T83A
VP1	capsid surface	Q108R	Q108R	-
VP1	capsid surface	Q110R	Q110R	Q110K
VP1	C-terminus	-	-	E202K
2C	-	K285Q	K285Q	K285Q

* According to Fry et al. [22].

**Table 3:** Growth comparison of Asia-1 Shamir and its mutants on receptor-deficient cell lines.

Cell Line	Virus Isolate			
	Asia-1 Shamir	#3 Asia-1	#8 Asia-1	#9 Asia-1
CHO-K1	negative	3.7 ± 0.5	3.7 ± 0.6	4.2 ± 0.6
CHO677	negative	2.4 ± 0.5	2.2 ± 0.2	3.8 ± 0.7

Values represent mean virus titers and standard deviation in log_10_ TCID_50_ per milliliter (TCID_50_/mL).

## Supplementary Materials

The following are available online at www.mdpi.com/1999-4915/9/8/231/s1, Table S1: Overview about the virus isolates used in this study and their passage history, Table S2: Passaging of FMDV Asia-1 Shamir on different BHK cell lines, Table S3: Additional primer mixes used for sequencing, Table S4: Virus passages of Asia-1 Shamir and other serotype Asia-1 isolates on BHK-2P, Table S5: Nucleotide and amino acid changes during passaging of Asia-1 in suspension cells.
