# Supplementary material for: Adaption of FMDV Asia-1 to Suspension Culture: Cell Resistance Is Overcome by Virus Capsid Alterations
Source: Viruses. 2017 Aug 18;9(8):231. doi: 10.3390/v9080231 (PMC5580488; doi:10.3390/v9080231)
Supplement: Supplementary file 1 [file viruses-09-00231-s001.zip › viruses-204040_final_supplementary.docx]

Supplementary Materials: Adaption of FMDV Asia-1 to Suspension Culture: Cell Resistance is Overcome by Virus Capsid Alterations

**Table S1.** Overview about the virus isolates used in this study and their passage history.

| **Virus Isolate** | **Passage History*** |
| --- | --- |
| Asia-1 Shamir/ISR/89 (original) | BHK6 (FLI) |
| #9 Asia-1 | + production BHK 5, BHK-2P 5 (FLI) |
| #8 Asia-1 | + BHK21-InVitrus 7, BHK-2P 9 (FLI) |
| #3 Asia-1 | + BHK21C13 7, BHK-2P 9 (FLI) |
| Asia TUR 6/2014 | BTY 1 (WRL), BHK 25 (FLI) |
| Asia TUR 6/2014-PT | BTY 1 (WRL), PT 18 (FLI) |
| Asia HKN 5/2005 | BHK 9 (FLI) |
| Asia PAK 5/2012 | BTY 1 (WRL), BHK 8 (FLI) |
| A_24_ Cruzeiro/BRA/55  (original) | CP 6 BHK 2 (WRL), BHK 4 (FLI) |
| A_24_-2P | + BHK-2P 20 (FLI) |
| O_1_ Manisa/TUR/69 | BHK 8 (WRL), BHK 8 (FLI) |

* Passage host/passage number (location). Cell lines: BHK: Baby hamster kidney; PT: Ovine cell line; CP: Cattle passage. BHK = adherent BHK21; BHK-2P, production BHK, BHK21-InVitrus,
BHK21C13 = suspension cell lines. WRL: FMD World Reference Laboratory, Pirbright, UK; FLI: Friedrich-Loeffler-Institut, Greifswald, Germany.

**Table S2.** Passaging of FMDV Asia-1 Shamir on different BHK cell lines.

Cells grown in monolayers:

| **Cell line and medium** | **Virus passage*** | **Infection volume** | **cytopathic effect (CPE)** |
| --- | --- | --- | --- |
|  |  |  |  |
| #1: adherent BHK21C13 in GMEM with 8.5% FBS | 1 | 50 µL | 100% after 24h |
|  |  | NC | no CPE after 24h |
|  | 2 | 30 µL | 100% after 24h |
|  |  | NC | no CPE after 24h |
|  | 3 | 10 µL | 100% after 24h |
|  |  | NC | no CPE after 24h |

Cells grown in suspension:

| **Cell line and medium** | **Virus passage*** | **Infection volume** | **Viability (%)** | **Total cells/mL (×10^5^)** | **Viable cells/mL (×10^5^)** |
| --- | --- | --- | --- | --- | --- |
|  |  |  |  |  |  |
| #2: BHK-2P in GMEM with 10% FBS | 1 | 5 mL | 77 | 9.9 | 7.7 |
|  |  | NC | 86 | 12.1 | 10.4 |
|  | 2 | 5 mL | 97 | 15.5 | 15.1 |
|  |  | NC | 100 | 17.1 | 17.0 |
|  | 3 | 10 mL | 93 | 11.6 | 10.8 |
|  |  | NC | 93 | 10.9 | 10.1 |
|  | 4 | 10 mL | 96 | 15.6 | 14.9 |
|  |  | NC | 94 | 17.1 | 16.1 |
|  | 5 | 10 mL | 92 | 12.2 | 11.2 |
|  |  | NC | 97 | 12.1 | 11.8 |
| #3: adherent BHK21C13 in Cellvento™ BHK-200 | 1 | 5 mL | 77 | 1.8 | 0.3 |
|  |  | NC | 86 | 1.9 | 1.8 |
|  | 2 | 5 mL | 54 | 4.7 | 2.5 |
|  |  | NC | 100 | 4.5 | 4.5 |
|  | 3 | 4 mL | 18 | 5.0 | 0.9 |
|  |  | NC | 89 | 3.4 | 3.0 |
| #4: BHK-2P in BHK-200  (high passage-p8) | 1 | 5 mL | 92 | 20.0 | 18.4 |
|  |  | NC | 97 | 17.0 | 16.5 |
|  | 2 | 5 mL | 97 | 29.9 | 29.0 |
|  |  | NC | 96 | 39.9 | 38.5 |
|  | 3 | 5 mL | 96 | 17.1 | 16.4 |
|  |  | NC | 97 | 14.2 | 13.8 |
|  | 4 | 5 mL | 95 | 13.7 | 13.0 |
|  |  | NC | 93 | 16.1 | 15.0 |
|  | 5 | 5 mL | 96 | 15.7 | 15.1 |
|  |  | NC | 99 | 15.0 | 14.8 |
|  | 6 | 5 mL | 73 | 13.0 | 9.6 |
|  |  | NC | 90 | 14.7 | 13.2 |

| **Cell line and medium** | **Virus passage*** | **Infection volume** | **Viability (%)** | **Total cells/mL (×10^5^)** | **Viable cells/mL (×10^5^)** |
| --- | --- | --- | --- | --- | --- |
| #5: BHK-2P short adaptation to BHK-200 (process 4) | 1 | 5 mL | 98 | 23.9 | 23.5 |
|  |  | NC | 97 | 22.6 | 21.9 |
|  | 2 | 5 mL | 97 | 46.3 | 45.1 |
|  |  | NC | 99 | 46.2 | 45.5 |
|  | 3 | 5 mL | 97 | 17.1 | 16.6 |
|  |  | NC | 98 | 23.2 | 22.7 |
|  | 4 | 5 mL | 98 | 23.0 | 22.6 |
|  |  | NC | 94 | 20.5 | 19.4 |
|  | 5 | 5 mL | 99 | 19.1 | 18.9 |
|  |  | NC | 99 | 37.2 | 36.7 |
| #6: BHK21-C in Cellvento™ BHK-200 | 1 | 5 mL | 97 | 22.1 | 21.4 |
|  |  | NC | 99 | 20.4 | 20.3 |
|  | 2 | 5 mL | 99 | 43.2 | 42.6 |
|  |  | NC | 98 | 52.1 | 51.2 |
|  | 3 | 5 mL | 98 | 20.7 | 20.7 |
|  |  | NC | 98 | 19.6 | 19.3 |
|  | 4 | 5 mL | 99 | 24.6 | 24.3 |
|  |  | NC | 99 | 32.6 | 32.4 |
|  | 5 | 5 mL | 98 | 23.7 | 23.3 |
|  |  | NC | 100 | 25.3 | 25.2 |
|  | 6 | 5 mL | 99 | 51.5 | 51.0 |
|  |  | NC | 98 | 48.2 | 47.4 |
| #7: BHK21- Hektor in Cellvento™ BHK-200 | 1 | 5 mL | 97 | 17.8 | 17.3 |
|  |  | NC | 99 | 20.3 | 20.1 |
|  | 2 | 5 mL | 97 | 34.1 | 32.9 |
|  |  | NC | 99 | 26.8 | 26.4 |
|  | 3 | 5 mL | 97 | 13.7 | 13.3 |
|  |  | NC | 98 | 16.0 | 15.6 |
|  | 4 | 5 mL | 94 | 19.0 | 17.9 |
|  |  | NC | 97 | 20.7 | 20.1 |
|  | 5 | 5 mL | 96 | 23.3 | 22.5 |
|  |  | NC | 98 | 20.4 | 20.0 |
|  | 6 | 5 mL | 95 | 33.5 | 32.0 |
|  |  | NC | 93 | 24.7 | 23.0 |

| **Cell line and medium** | **Virus passage*** | **Infection volume** | **Viability (%)** | **Total cells/mL (×10^5^)** | **Viable cells/mL (×10^5^)** |
| --- | --- | --- | --- | --- | --- |
| #8: BHK21- InVitrus in Cellvento™ BHK-200 | 1 | 5 mL | 88 | 17.5 | 15.3 |
|  |  | NC | 98 | 21.6 | 21.1 |
|  | 2 | 5 mL | 84 | 28.9 | 24.3 |
|  |  | NC | 98 | 32.7 | 32.2 |
|  | 3 | 5 mL | 67 | 10.4 | 6.9 |
|  |  | NC | 98 | 15.8 | 15.6 |
|  | 4 | 5 mL | 9 | 11.4 | 1.0 |
|  |  | NC | 96 | 14.8 | 14.2 |
|  | 5 | 3 mL | 5 | 7.4 | 0.3 |
|  |  | NC | 96 | 23.9 | 22.9 |
| #9: production BHK in Cellvento™ BHK-200 | 1 | 5 mL | 84 | 27.2 | 22.9 |
|  |  | NC | 96 | 18.8 | 18.1 |
|  | 2 | 5 mL | 56 | 8.6 | 4.8 |
|  |  | NC | 86 | 7.3 | 6.3 |
|  | 3 | 5 mL | 52 | 17.3 | 9.0 |
|  |  | NC | 92 | 13.2 | 12.2 |
|  | 4 | 4 mL | 40 | 49.6 | 21.1 |
|  |  | NC | 97 | 15.3 | 14.9 |

*Cells have different total numbers of passages due to different growth properties.

**Table S3.** Additional primer mixes used for sequencing.

| **Primer** | **Primer Sequence 5'–3'** | **Locations* 5'–3'** | **Amplicon size** |
| --- | --- | --- | --- |
| VP3-2835F | TCG ACG TGT CCC TCG C | 2835-2851 | 366 bp |
| VP3-3232R | AAGTCTTTGCCGGCG | 3217-3232 |  |
| VP1-3165F | ATCAGATCACCCACGG | 3165-3181 | 451 bp |
| VP1-3649R | GTTGCCAGCACACGATG | 3632-3649 |  |
| 3D-7320F | GTTGCAACCCTGATGT | 7320-7336 | 761 bp |
| 3D-8113R | TTCTGCCAATTGCGAC | 8097-8113 |  |

* Location in genome of JF739177.

**Table S4.** Virus passages of Asia-1 Shamir and other serotype Asia-1 isolates on BHK-2P.

First adaption attempt of Asia-1 Shamir

| **Passage No.** | **cell number (cells/mL)** | **infection volume (mL)** | **cell viability 24 hpi** |
| --- | --- | --- | --- |
| 0 | BHK164 (adherent) | 50 µL in 10 mL | 100% CPE |
| 1 | 8.3 ×10^5^ (undefined) | 1.35 mL in 30 mL | 99% |
| 2 | 4.9 ×10^6^ (undefined) | 1.3 | 100% |
| 3 | 4.6 ×10^6^ (undefined) | 10.0 | 98% |
| 4 | 1.0 ×10^6^ | 5.0 | 97% |
| 5 | 1.0 ×10^6^ | 5.0 | 98% |
| 6 | 1.0 ×10^6^ | 10.0 | 98% |
| Infection control | BHK164 (adherent) | 1 mL of P6 | 0% CPE |

Second adaption attempt of Asia-1 Shamir

| **Passage No.** | **cell number (cells/mL)** | **infection volume (mL)** | **cell viability 24 hpi** |
| --- | --- | --- | --- |
| 0 | BHK164 (adherent) | 50 µL in 10 mL | 100% CPE |
| 1 | 1.0 ×10^6^ | 5.0 | 98% |
| 2 | 1.0 ×10^6^ | 15.0 | 97% |
| 3 | 1.0 ×10^6^ | 15.0 | 96% |
| 4 | 1.0 ×10^6^ | 15.0 | 96% |
| 5 | 1.0 ×10^6^ | 15.0 | 97% |
| 6 | 1.0 ×10^6^ | 15.0 | 92% |
| 7 | 1.0 ×10^6^ | 15.0 | 99% |
| 8 | 1.0 ×10^6^ | 15.0 | 96% |
| 9 | 1.0 ×10^6^ | 15.0 | 97% |
| Infection control | BHK164 (adherent) | 1 mL of P9 | 0% CPE |

Third adaption attempt of Asia-1 Shamir

| **Passage No.** | **cell number (cells/mL)** | **infection volume (mL)** | **cell viability 24 hpi** |
| --- | --- | --- | --- |
| 0 | BHK179 (adherent) | 50 µL in 10 mL | 100% CPE |
| 1 | 1.0 ×10^6^ | 10.0 | 99% |
| 2 | 1.0 ×10^6^ | 15.0 | 99% |
| 3 | 1.0 ×10^6^ | 15.0 | 96% |
| 4 | 1.0 ×10^6^ | 15.0 | 98% |
| 5 | 1.0 ×10^6^ | 15.0 | 96% |
| 6 | 1.0 ×10^6^ | 15.0 | 99% |
| Infection control | LFBKαvβ6 (adherent) | 50 µL of P5 | 0% CPE |

Adaption attempts of other serotype Asia-1 strains

Asia-1 TUR 6/2014

| **Passage No.** | **cell number (cells/mL)** | **infection volume (mL)** | **cell viability 24 hpi** |
| --- | --- | --- | --- |
| 0 | BHK179 (adherent) | 50 µL in 10 mL | 100% CPE |
| 1 | 1.0 ×10^6^ | 10.0 | 98% |
| 2 | 1.0 ×10^6^ | 15.0 | 100% |
| 3 | 1.0 ×10^6^ | 15.0 | 99% |
| 4 | 1.0 ×10^6^ | 15.0 | 95% |
| 5 | 1.0 ×10^6^ | 15.0 | 99% |
| Infection control | LFBKαvβ6 (adherent) | 50 µL of P4 | 0% CPE |

Asia-1 TUR 6/2014-PT

| **Passage No.** | **cell number (cells/mL)** | **infection volume (mL)** | **cell viability 24 hpi** |
| --- | --- | --- | --- |
| 0 | BHK179 (adherent) | 50 µL in 10 mL | 100% CPE |
| 1 | 1.0 ×10^6^ | 10.0 | 99% |
| 2 | 1.0 ×10^6^ | 15.0 | 100% |
| 3 | 1.0 ×10^6^ | 15.0 | 99% |
| 4 | 1.0 ×10^6^ | 15.0 | 100% |
| 5 | 1.0 ×10^6^ | 15.0 | 99% |
| Infection control | LFBKαvβ6 (adherent) | 50 µL of P4 | 0% CPE |

Asia-1 HKN 5/2005

| **Passage No.** | **cell number (cells/mL)** | **infection volume (mL)** | **cell viability 24 hpi** |
| --- | --- | --- | --- |
| 0 | BHK179 (adherent) | 50 µL in 10 mL | 100% CPE |
| 1 | 1.0 ×10^6^ | 10.0 | 99% |
| 2 | 1.0 ×10^6^ | 15.0 | 99% |
| 3 | 1.0 ×10^6^ | 15.0 | 97% |
| 4 | 1.0 ×10^6^ | 15.0 | 99% |
| 5 | 1.0 ×10^6^ | 15.0 | 95% |
| 6 | 1.0 ×10^6^ | 15.0 | 99% |
| Infection control | LFBKαvβ6 (adherent) | 50 µL of P5 | 0% CPE |

Asia-1 PAK 5/2012

| **Passage No.** | **cell number (cells/mL)** | **infection volume (mL)** | **cell viability 24 hpi** |
| --- | --- | --- | --- |
| 0 | BHK179 (adherent) | 50 µL in 10 mL | 100% CPE |
| 1 | 1.0 ×10^6^ | 10.0 | 99% |
| 2 | 1.0 ×10^6^ | 15.0 | 99% |
| 3 | 1.0 ×10^6^ | 15.0 | 99% |
| 4 | 1.0 ×10^6^ | 15.0 | 99% |
| 5 | 1.0 ×10^6^ | 15.0 | 96% |
| 6 | 1.0 ×10^6^ | 15.0 | 99% |
| Infection control | LFBKαvβ6 (adherent) | 50 µL of P5 | 0% CPE |

**Table S5.** Nucleotide and amino acid changes during passaging of Asia-1 in suspension cells.

| **Genome Region** | **Virus Isolate** | | | **AA Change** | **Change in Charge/Polarity** |
| --- | --- | --- | --- | --- | --- |
|  | **#3 Asia-1** | **#8 Asia-1** | **#9 Asia-1** |  |  |
| IRES | T1028Y | - | - |  |  |
| Leader | C1195T | | | no | no |
| VP3 | - | - | G2783A | yes, E to K | negative to positive charge |
| VP1 | - | - | A3512G | yes, T to A | polar uncharged to hydrophobic side chain |
| VP1 | A3588G | | - | yes, Q to R | polar uncharged side chain to positive charge |
| VP1 | - | - | C3593A | yes, Q to K | polar uncharged side chain to positive charge |
| VP1 | A3594G | | - | yes, Q to R | polar uncharged side chain to positive charge |
| VP1 | - | - | G3869A | yes, E to K | negative to positive charge |
| 2C | A4413G | | | yes, K to R | no |
| 2C | A5261C | | | yes, K to Q | positive charge to polar uncharged side chain |
| 3A | A5622G | - | - | yes, D to G | negative charge to uncharged |
| 3C | A6449G | - | - | yes, I to V | no |
| 3D | - | - | T7339C | no | no |

Mutations that are discussed in detail in the paper are shown in italics. The remaining mutations are included here for the sake of completeness, but are considered irrelevant for the purposes of this study.
