# Supplementary material for: Adaption of FMDV Asia-1 to Suspension Culture: Cell Resistance Is Overcome by Virus Capsid Alterations
Source: Viruses. 2017 Aug 18;9(8):231. doi: 10.3390/v9080231 (PMC5580488; doi:10.3390/v9080231)
Supplement: Supplementary file 1 [file viruses-09-00231-s001.zip › viruses-204040_final_supplementary.pdf]

Supplementary Materials: Adaption of FMDV Asia-1 to Suspension Culture: Cell Resistance is Overcome by Virus Capsid Alterations

Table S1. Overview about the virus isolates used in this study and their passage history.

Virus Isolate	Passage History*
Asia-1 Shamir/ISR/89 (original)	BHK6 (FLI)
#9 Asia-1	+ production BHK 5, BHK-2P 5 (FLI)
#8 Asia-1	+ BHK21-InVitrus 7, BHK-2P 9 (FLI)
#3 Asia-1	+ BHK21C13 7, BHK-2P 9 (FLI)
Asia TUR 6/2014	BTY 1 (WRL), BHK 25 (FLI)
Asia TUR 6/2014-PT	BTY 1 (WRL), PT 18 (FLI)
Asia HKN 5/2005	BHK 9 (FLI)
Asia PAK 5/2012	BTY 1 (WRL), BHK 8 (FLI)
A_{24} Cruzeiro/BRA/55 (original)	CP 6 BHK 2 (WRL), BHK 4 (FLI)
A_{24}-2P	+ BHK-2P 20 (FLI)
O_1 Manisa/TUR/69	BHK 8 (WRL), BHK 8 (FLI)

* Passage host/passage number (location). Cell lines: BHK: Baby hamster kidney; PT: Ovine cell line; CP: Cattle passage. BHK = adherent BHK21; BHK-2P, production BHK, BHK21-InVitrus, BHK21C13 = suspension cell lines. WRL: FMD World Reference Laboratory, Pirbright, UK; FLI: Friedrich-Loeffler-Institut, Greifswald, Germany.

Table S2. Passaging of FMDV Asia-1 Shamir on different BHK cell lines.

Cells grown in monolayers:

Cell line and medium	Virus passage*	Infection volume	cytopathic effect (CPE)
#1: adherent BHK21C13 in GMEM with 8.5% FBS	1	50 µL	100% after 24h
		NC	no CPE after 24h
	2	30 µL	100% after 24h
		NC	no CPE after 24h
	3	10 µL	100% after 24h
		NC	no CPE after 24h

Cells grown in suspension:

Cell line and medium	Virus passage*	Infection volume	Viability (%)	Total cells/mL ($\times10^5$)	Viable cells/mL ($\times10^5$)
#2: BHK-2P in GMEM with 10% FBS	1	5 mL	77	9.9	7.7
		NC	86	12.1	10.4
	2	5 mL	97	15.5	15.1
		NC	100	17.1	17.0
	3	10 mL	93	11.6	10.8
		NC	93	10.9	10.1
	4	10 mL	96	15.6	14.9
		NC	94	17.1	16.1
	5	10 mL	92	12.2	11.2
		NC	97	12.1	11.8
#3: adherent BHK21C13 in Cellvento™ BHK-200	1	5 mL	77	1.8	0.3
		NC	86	1.9	1.8
	2	5 mL	54	4.7	2.5
		NC	100	4.5	4.5
	3	4 mL	18	5.0	0.9
		NC	89	3.4	3.0
#4: BHK-2P in BHK-200 (high passage-p8)	1	5 mL	92	20.0	18.4
		NC	97	17.0	16.5
	2	5 mL	97	29.9	29.0
		NC	96	39.9	38.5
	3	5 mL	96	17.1	16.4
		NC	97	14.2	13.8
	4	5 mL	95	13.7	13.0
		NC	93	16.1	15.0
	5	5 mL	96	15.7	15.1
		NC	99	15.0	14.8
	6	5 mL	73	13.0	9.6

NC	90	14.7	13.2

Cell line and medium	Virus passage*	Infection volume	Viability (%)	Total cells/mL (×10^5)	Viable cells/mL (×10^5)
#5: BHK-2P short adaptation to BHK-200 (process 4)	1	5 mL	98	23.9	23.5
		NC	97	22.6	21.9
	2	5 mL	97	46.3	45.1
		NC	99	46.2	45.5
	3	5 mL	97	17.1	16.6
		NC	98	23.2	22.7
	4	5 mL	98	23.0	22.6
		NC	94	20.5	19.4
	5	5 mL	99	19.1	18.9
		NC	99	37.2	36.7
#6: BHK21-C in Cellvento™ BHK-200	1	5 mL	97	22.1	21.4
		NC	99	20.4	20.3
	2	5 mL	99	43.2	42.6
		NC	98	52.1	51.2
	3	5 mL	98	20.7	20.7
		NC	98	19.6	19.3
	4	5 mL	99	24.6	24.3
		NC	99	32.6	32.4
	5	5 mL	98	23.7	23.3
		NC	100	25.3	25.2
	6	5 mL	99	51.5	51.0
		NC	98	48.2	47.4
#7: BHK21- Hektor in Cellvento™ BHK-200	1	5 mL	97	17.8	17.3
		NC	99	20.3	20.1
	2	5 mL	97	34.1	32.9
		NC	99	26.8	26.4
	3	5 mL	97	13.7	13.3
		NC	98	16.0	15.6
	4	5 mL	94	19.0	17.9
		NC	97	20.7	20.1
	5	5 mL	96	23.3	22.5
		NC	98	20.4	20.0
	6	5 mL	95	33.5	32.0
		NC	93	24.7	23.0

Cell line and medium	Virus passage*	Infection volume	Viability (%)	Total cells/mL (×10^5)	Viable cells/mL (×10^5)
#8: BHK21- InVitrus in Cellvento™ BHK-200	1	5 mL	88	17.5	15.3
		NC	98	21.6	21.1
	2	5 mL	84	28.9	24.3
		NC	98	32.7	32.2
	3	5 mL	67	10.4	6.9
		NC	98	15.8	15.6
	4	5 mL	9	11.4	1.0
		NC	96	14.8	14.2
	5	3 mL	5	7.4	0.3
		NC	96	23.9	22.9
#9: production BHK in Cellvento™ BHK-200	1	5 mL	84	27.2	22.9
		NC	96	18.8	18.1
	2	5 mL	56	8.6	4.8
		NC	86	7.3	6.3
	3	5 mL	52	17.3	9.0
		NC	92	13.2	12.2
	4	4 mL	40	49.6	21.1
		NC	97	15.3	14.9

*Cells have different total numbers of passages due to different growth properties.

Table S3. Additional primer mixes used for sequencing.

Primer	Primer Sequence 5'–3'	Locations* 5'–3'	Amplicon size
VP3-2835F	TCG ACG TGT CCC TCG C	2835-2851	366 bp
VP3-3232R	AAGTCTTTGCCGGCG	3217-3232	
VP1-3165F	ATCAGATCACCCACGG	3165-3181	451 bp
VP1-3649R	GTTGCCAGCACACGATG	3632-3649	
3D-7320F	GTTGCAACCCTGATGT	7320-7336	761 bp
3D-8113R	TTCTGCCAATTGCGAC	8097-8113	

* Location in genome of JF739177.

Table S4. Virus passages of Asia-1 Shamir and other serotype Asia-1 isolates on BHK-2P.

First adaption attempt of Asia-1 Shamir

Passage No.	cell number (cells/mL)	infection volume (mL)	cell viability 24 hpi
0	BHK164 (adherent)	50 µL in 10 mL	100% CPE
1	8.3×10^5 (undefined)	1.35 mL in 30 mL	99%
2	4.9×10^6 (undefined)	1.3	100%
3	4.6×10^6 (undefined)	10.0	98%
4	1.0×10^6	5.0	97%
5	1.0×10^6	5.0	98%
6	1.0×10^6	10.0	98%
Infection control	BHK164 (adherent)	1 mL of P6	0% CPE

Second adaption attempt of Asia-1 Shamir

Passage No.	cell number (cells/mL)	infection volume (mL)	cell viability 24 hpi
0	BHK164 (adherent)	50 µL in 10 mL	100% CPE
1	1.0×10^6	5.0	98%
2	1.0×10^6	15.0	97%
3	1.0×10^6	15.0	96%
4	1.0×10^6	15.0	96%
5	1.0×10^6	15.0	97%
6	1.0×10^6	15.0	92%
7	1.0×10^6	15.0	99%
8	1.0×10^6	15.0	96%
9	1.0×10^6	15.0	97%
Infection control	BHK164 (adherent)	1 mL of P9	0% CPE

Third adaption attempt of Asia-1 Shamir

Passage No.	cell number (cells/mL)	infection volume (mL)	cell viability 24 hpi
0	BHK179 (adherent)	50 µL in 10 mL	100% CPE
1	1.0×10^6	10.0	99%
2	1.0×10^6	15.0	99%
3	1.0×10^6	15.0	96%
4	1.0×10^6	15.0	98%
5	1.0×10^6	15.0	96%
6	1.0×10^6	15.0	99%
Infection control	LFBKαvβ6 (adherent)	50 µL of P5	0% CPE

Adaption attempts of other serotype Asia-1 strains

Asia-1 TUR 6/2014

Passage No.	cell number (cells/mL)	infection volume (mL)	cell viability 24 hpi
0	BHK179 (adherent)	50 µL in 10 mL	100% CPE
1	1.0×10^6	10.0	98%
2	1.0×10^6	15.0	100%
3	1.0×10^6	15.0	99%
4	1.0×10^6	15.0	95%
5	1.0×10^6	15.0	99%
Infection control	LFBKαvβ6 (adherent)	50 µL of P4	0% CPE

Asia-1 TUR 6/2014-PT

Passage No.	cell number (cells/mL)	infection volume (mL)	cell viability 24 hpi
0	BHK179 (adherent)	50 µL in 10 mL	100% CPE
1	1.0×10^6	10.0	99%
2	1.0×10^6	15.0	100%
3	1.0×10^6	15.0	99%
4	1.0×10^6	15.0	100%
5	1.0×10^6	15.0	99%
Infection control	LFBKαvβ6 (adherent)	50 µL of P4	0% CPE

Asia-1 HKN 5/2005

Passage No.	cell number (cells/mL)	infection volume (mL)	cell viability 24 hpi
0	BHK179 (adherent)	50 µL in 10 mL	100% CPE
1	1.0×10^6	10.0	99%
2	1.0×10^6	15.0	99%
3	1.0×10^6	15.0	97%
4	1.0×10^6	15.0	99%
5	1.0×10^6	15.0	95%
6	1.0×10^6	15.0	99%
Infection control	LFBKαvβ6 (adherent)	50 µL of P5	0% CPE

Asia-1 PAK 5/2012

Passage No.	cell number (cells/mL)	infection volume (mL)	cell viability 24 hpi
0	BHK179 (adherent)	50 µL in 10 mL	100% CPE
1	1.0×10^6	10.0	99%
2	1.0×10^6	15.0	99%
3	1.0×10^6	15.0	99%
4	1.0×10^6	15.0	99%
5	1.0×10^6	15.0	96%
6	1.0×10^6	15.0	99%
Infection control	LFBKαvβ6 (adherent)	50 µL of P5	0% CPE

Table S5. Nucleotide and amino acid changes during passaging of Asia-1 in suspension cells.

Genome	Virus Isolate			AA	Change in Charge/Polarity
Region	#3 Asia-1	#8 Asia-1	#9 Asia-1	Change	
IRES	T1028Y	-	-		
Leader		C1195T		no	no
VP3	-	-	G2783A	yes, E to K	negative to positive charge
VP1	-	-	A3512G	yes, T to A	polar uncharged to hydrophobic side chain
VP1	A3588G		-	yes, Q to R	polar uncharged side chain to positive charge
VP1	-	-	C3593A	yes, Q to K	polar uncharged side chain to positive charge
VP1	A3594G		-	yes, Q to R	polar uncharged side chain to positive charge
VP1	-	-	G3869A	yes, E to K	negative to positive charge
2C		A4413G		yes, K to R	no
2C		A5261C		yes, K to Q	positive charge to polar uncharged side chain
3A	A5622G	-	-	yes, D to G	negative charge to uncharged
3C	A6449G	-	-	yes, I to V	no
3D	-	-	T7339C	no	no

Mutations that are discussed in detail in the paper are shown in italics. The remaining mutations are included here for the sake of completeness, but are considered irrelevant for the purposes of this study.
